# Topic-Modeling Guided Semantic Clustering for Enhancing CNN-Based Image Classification Using Scale-Invariant Feature Transform and Block Gabor Filtering

**DOI:** 10.3390/jimaging12020070

**Published:** 2026-02-09

**Authors:** Natthaphong Suthamno, Jessada Tanthanuch

**Affiliations:** School of Mathematical Sciences and Geoinformatics, Institute of Science, Suranaree University of Technology, Nakhon Ratchasima 30000, Thailand; nutthapong.su@gmail.com

**Keywords:** image clustering, Latent Dirichlet Allocation, convolutional neural network, Block Gabor Filter, Scale-Invariant Feature Transform

## Abstract

This study proposes a topic-modeling guided framework that enhances image classification by introducing semantic clustering prior to CNN training. Images are processed through two key-point extraction pipelines: Scale-Invariant Feature Transform (SIFT) with Sobel edge detection and Block Gabor Filtering (BGF), to obtain local feature descriptors. These descriptors are clustered using K-means to build a visual vocabulary. Bag of Words histograms then represent each image as a visual document. Latent Dirichlet Allocation is applied to uncover latent semantic topics, generating coherent image clusters. Cluster-specific CNN models, including AlexNet, GoogLeNet, and several ResNet variants, are trained under identical conditions to identify the most suitable architecture for each cluster. Two topic guided integration strategies, the Maximum Proportion Topic (MPT) and the Weight Proportion Topic (WPT), are then used to assign test images to the corresponding specialized model. Experimental results show that both the SIFT-based and BGF-based pipelines outperform non-clustered CNN models and a baseline method using Incremental PCA, K-means, Same-Cluster Prediction, and unweighted Ensemble Voting. The SIFT pipeline achieves the highest accuracy of 95.24% with the MPT strategy, while the BGF pipeline achieves 93.76% with the WPT strategy. These findings confirm that semantic structure introduced through topic modeling substantially improves CNN classification performance.

## 1. Introduction

Image classification remains a fundamental task in computer vision, underpinning applications such as medical image analysis, scene understanding, and content-based image retrieval. Recent advances in deep learning, particularly convolutional neural networks (CNNs), have dramatically improved classification accuracy. However, conventional CNN training typically treats all images as independent, equally weighted samples, which may fail to capture the latent semantic structure inherent in complex datasets [1]. Images often contain heterogeneous visual content, where local patterns and contextual cues contribute unequally to semantic categories. Addressing this challenge, topic-modeling techniques such as Latent Dirichlet Allocation (LDA) offer a promising approach to discover underlying semantic structures and group images into coherent clusters.

Latent Dirichlet Allocation (LDA), originally developed for text analysis, has been adapted for image clustering and classification [2]. Pradheep and Jayaraman (2005) pioneered applying LDA to images by converting image segments into visual words using k-means clustering on color histograms, representing each image as a mixture of latent topics [3]. Experiments on both small and moderately sized datasets demonstrated that LDA-based representations could produce meaningful clusters reflecting visual similarities, with performance improving on larger datasets. In the same year, Fei-Fei and Perona (2005) proposed a Bayesian hierarchical model inspired by LDA for unsupervised natural scene categorization [4]. Their method treated images as collections of local patches (codewords) and introduced latent “themes” as intermediate semantic constructs summarizing parts of a scene. Using variational inference and Expectation-Maximization (EM), the model effectively categorized scenes without manual labeling. Notably, 128-dimensional Scale-Invariant Feature Transform (SIFT) features outperformed 11 × 11-pixel patches, demonstrating the feasibility of discovering meaningful intermediate structures in an unsupervised manner (approximately 65% accuracy in some configurations).

Philbin et al. (2011) introduced Geometric LDA (gLDA), which incorporated geometric consistency among local image features through a matching graph [5]. By enforcing geometric constraints during topic discovery, gLDA improved unsupervised object discovery in large-scale image collections and extracted coherent objects or scene parts. The approach scaled well and produced interpretable patterns corresponding to meaningful visual structures. In 2014, Gabor filters were used in combination with Fuzzy C-Means (FCM) clustering to improve MRI brain image segmentation [6]. Adaptive 2D Gabor filters at multiple scales and orientations extracted robust texture features, which were then clustered using FCM to segment main brain tissues. This approach showed superior noise resilience and segmentation accuracy compared with conventional methods. In 2016, Gabor filtering was integrated with LDA to extract texture and local statistical features for breast histopathological image retrieval [7]. Using over 8000 image patches, this method achieved a high retrieval precision (approximately 0.954), demonstrating that Gabor-based features could capture meaningful patterns in tissue images suitable for large-scale retrieval tasks.

In 2019, several LDA-based studies extended applications to both medical and general image datasets. For multimodal medical image retrieval, one study combined LDA on SIFT-derived visual words with textual features, while another introduced a topic-and-location model that incorporated the spatial locations of visual words, improving retrieval from large and anatomically diverse medical image datasets [8,9]. Using this method, the authors reported a mean average precision (MAP) of 86.74% on a multimodal dataset and a top-ten retrieval precision of 97.5% on the IRMA 2009 dataset, highlighting the value of combining semantic topics with spatial context. Also in 2019, Wang et al. proposed an unsupervised framework for semi-automated image annotation and data curation using image clustering and topic modeling [10]. The approach employed LDA alongside SIFT features and pre-trained CNN embeddings from ImageNet to group unlabeled images into meaningful clusters and latent topics. Images were represented using local SIFT features and CNN embeddings to capture content at different levels. The framework effectively organized images into semantically coherent clusters without supervision, highlighting that LDA works best with local feature representations such as SIFT, whereas global features like ResNet18 embeddings may be less suitable. This study emphasizes that local descriptors are more compatible with topic-modeling for unsupervised clustering tasks.

Beyond topic modeling, advances in clustering methodologies continue to contribute to the understanding and organization of high-dimensional image data. In 2025, recent work on the Separation-Optimized Number of Smart Clusters (SONSC) framework introduces an adaptive, validity-index-driven approach for determining the optimal number of clusters without manual parameter tuning [11]. SONSC employs the Improved Separation Index (ISI) to jointly assess intra-cluster compactness and inter-cluster separability, enabling reliable unsupervised grouping across diverse datasets. Although not directly related to topic modeling, SONSC reinforces the broader trend toward adaptive, interpretable clustering techniques that enhance the organization and analysis of heterogeneous visual data. Additionally, recent work on medical image classification for brain tumor analysis by Yahya Dogan proposes a hybrid framework that integrates a pruning-enhanced EfficientNetV2B3 model with traditional machine learning classifiers and metaheuristic-based feature selection techniques, including Genetic Algorithms, Particle Swarm Optimization, and the Lion Optimization Algorithm [12]. This approach provides a computationally efficient and interpretable solution, making it well suited for deployment in real-time and resource-constrained clinical environments.

Taken together, these studies indicate that:

- LDA-based topic-modeling effectively captures latent semantic structures in images, particularly when combined with local features such as SIFT or Gabor descriptors;
- The incorporation of spatial, geometric, or multimodal information can further enhance the quality of clustering and improve subsequent classification performance;
- Integrating semantic clustering with deep learning architectures presents a feasible approach for leveraging topic distributions to guide cluster-specific model training and prediction.

Despite the success of LDA-based clustering and deep CNNs, few studies have explored the combination of topic-modeling with cluster-specific CNN training for improved image classification. Most existing approaches either use global feature embeddings, which may not capture fine-grained local structures, or rely on simple ensemble mechanisms without semantic guidance. Furthermore, adaptive integration of multiple cluster-specific models using topic distributions has not been fully investigated. Addressing these gaps could improve both interpretability and predictive performance in image classification tasks, particularly for heterogeneous datasets.

This study proposes a topic-modeling-based framework for image classification that integrates LDA-guided semantic clustering with cluster-specific CNN models. The key contributions are:

- Semantic clustering of images: Images are transformed into visual documents using local descriptors, such as SIFT or Block Gabor Filtering (BGF), and LDA identifies latent topics to create coherent clusters;
- Cluster-specific CNN training: CNNs are trained separately on each topic cluster, enabling specialization on semantically homogeneous subsets;
- Topic-guided model integration: Two strategies—Maximum Proportion Topic and Weight Proportion Topic—leverage document-topic distributions to select or weight predictions across clusters;
- Comprehensive evaluation: The framework is evaluated against baseline non-clustered CNN training and ensemble methods, demonstrating superior accuracy and robustness across different feature extraction techniques.

This work demonstrates that combining semantic topic modeling with deep learning can improve image classification by providing structured, interpretable partitions that guide both training and inference, offering a promising direction for future large-scale and heterogeneous datasets.

## 2. Materials

This study evaluates the proposed framework using a medical imaging dataset characterized by substantial visual variability despite representing the same disease category. Specifically, brain tumor MRI images were selected, as such data exhibit diverse tumor appearances in terms of shape, location, and texture. This diversity makes the dataset well suited for examining whether topic modeling can effectively capture meaningful semantic patterns related to tumor characteristics.

The dataset used in this study was obtained from publicly available data on Kaggle, namely the Brain Tumors 256 × 256, consisting of 3096 MRI images [13]. The dataset, uploaded by Thomas Dubail in 2023 under the CC0 Public Domain license, includes 438 normal (N) images and 2658 tumor images (comprising 901 glioma (G), 913 meningioma (M), and 844 pituitary (P) tumor cases). All images were resized to a resolution of 256 × 256 pixels while maintaining their original aspect ratios to ensure consistency and preserve image detail.

**Figure 1:**
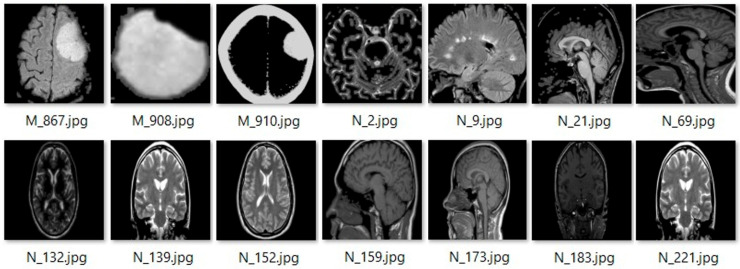
Examples of images excluded prior to the preprocessing stage.

In this study, images that were not in a head shape or exhibited abnormal head shapes, as illustrated in Figure 1, were removed during data preprocessing, resulting in the exclusion of 6 glioma, 21 meningioma, 7 pituitary tumor, and 21 normal images. The remaining images were then divided into training and testing sets at an 80:20 ratio, comprising 2432 training images and 609 testing images.

## 3. Methodology

**Figure 2:**
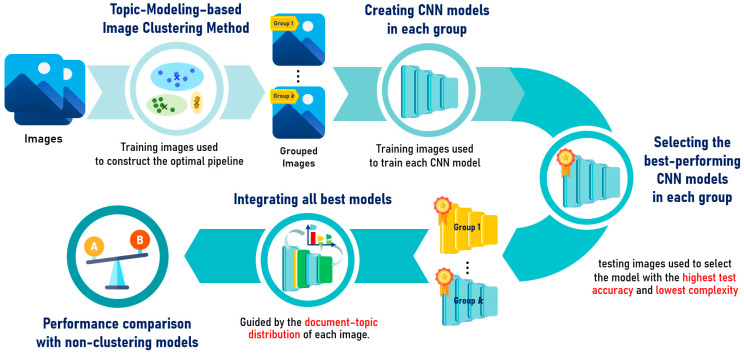
The overall workflow of proposed method.

This work proposes a hybrid approach that integrates topic modeling with CNN models for image classification. The overall workflow of this approach is illustrated in Figure 2. To ensure a fair and comprehensive performance assessment, the proposed method is compared against a traditional clustering approach, which serves as the baseline. This section provides a full description of both the baseline and the proposed pipelines.

### 3.1. Baseline Clustering

**Figure 3:**
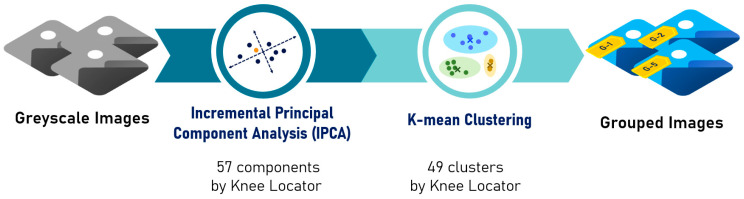
Workflow of the baseline clustering method.

In this study, a baseline was constructed to evaluate clustering performance by comparing the proposed method with a traditional approach using K-means and Incremental Principal Component Analysis (IPCA), an extension of PCA for large-scale or streaming datasets [14]. The overall structure of the baseline is presented in Figure 3.

**Figure 4:**
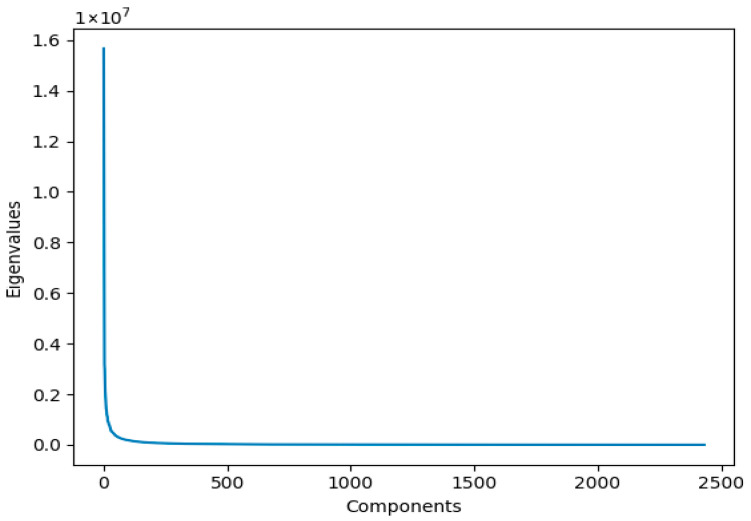
The curve plotting the relationship between the number of components and their eigenvalues.

The preprocessing stage involved converting the RGB images into grayscale representations. Thereafter, IPCA was applied to reduce the dimensionality of the data to 57 components. This number was identified as optimal based on the knee point in the curve plotting the number of components (ranging from 1 to 2432—the number of training images) against the eigenvalues of the components as shown in Figure 4.

**Figure 5:**
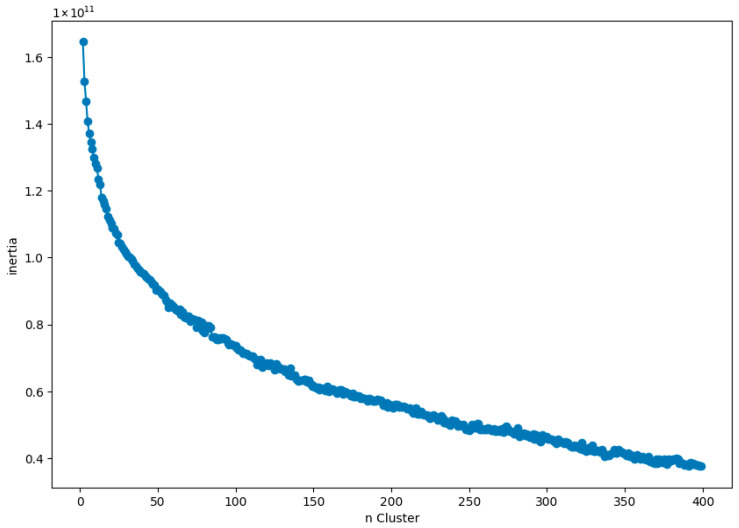
The plot between the number of clusters and the inertia.

After dimensionality reduction, K-means algorithm was applied to cluster the images based on the feature components obtained from IPCA. The relationship between the number of clusters (ranging from 2 to 400) and the inertia—defined as the sum of squared distances of samples from their nearest centroid—was visualized as shown in Figure 5. The optimal number of clusters was found to be 49 using the knee point of the graph determined by the Knee Locator library. Subsequently, the grayscale images were divided into 49 clusters. The training images within each cluster were then utilized to train multiple convolutional neural network models, including AlexNet, GoogLeNet, and several ResNet variants, in order to determine the model that achieved the highest accuracy on the test image set.

**Figure 6:**
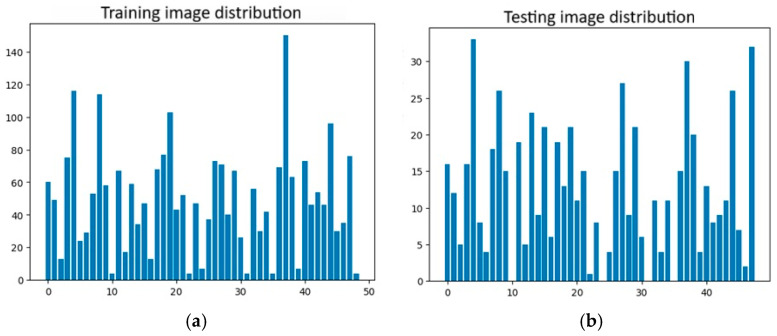
The distribution of the number of images in each cluster in the training (**a**) and testing (**b**) sets.

However, several clusters contained relatively few images in both the training and test sets as illustrated Figure 6a,b. To ensure reliable model training, only clusters with a number of images exceeding the average across all clusters in both sets were considered for model construction and optimal model selection. The average numbers of training and testing images across all clusters were 49.63 and 13.84, respectively. After filtering out small clusters, a total of 19 clusters remained, containing 1490 training images and 407 testing images. The details of each cluster are presented in Table 1.

### 3.2. Latent Dirichlet Allocation

LDA, first introduced by Blei, Ng and Jordan in 2003, is a generative probabilistic model widely used for topic modeling in NLP [2]. This approach uncovers the latent topic structure in large text corpora, enabling tasks such as document clustering, document summarization, and document recommendation systems.

The assumptions of LDA state that each document in a collection is represented as a mixture of multiple latent topics, and each topic is characterized by a distribution over words. The proportion of topics within a document is referred to as the document–topic distribution, whereas the distribution of words within a topic is known as the topic–word distribution. There are two hyperparameters, α and β, which control the distribution of topics per document and words per topic, respectively. Since the document–topic distribution follows a Dirichlet distribution, where $\alpha_{i}\;<\;1$ for all i = 1, 2,…,k, Most documents tend to contain a dominant topic whose proportion is significantly higher than those of the other topics. If $\alpha_{i}\;=\;1$ for all i = 1, 2,…,k, the distribution of topics becomes uniform and random. In case of $\alpha_{i}\;>\;1$ for all i = 1, 2,…,k, the topic proportions are more evenly distributed across topics. The topic–word distribution follows a similar pattern to the document–topic distribution [15].

As a generative model, LDA can also generate documents based on the document–topic and topic–word distributions of the corpus. However, the exact distributional properties of these two distributions are unknown. Therefore, a Bayesian inference method is employed to estimate them by approximating the distribution characteristics of words or observed variables.

### 3.3. Coherence Score

In the context of topic modeling, the coherence score measures how interpretable and meaningful topics are to humans [16]. It quantifies the semantic relatedness or co-occurrence of top words within a topic, reflecting the overall coherence of the topic based on the test data.

Among existing coherence metrics, the C_v_ score is particularly notable due to its high correlation with human evaluations of topic interpretability. It computes topic coherence by measuring word co-occurrence within a sliding window across a reference corpus and integrating Normalized Pointwise Mutual Information (NPMI) with cosine similarity calculations [17]. The score typically ranges between 0 and 1, with higher values indicating more semantically coherent topics and values closer to 0 reflecting weak topic interpretability.

### 3.4. Topic-Modeling-Based Clustering

**Figure 7:**
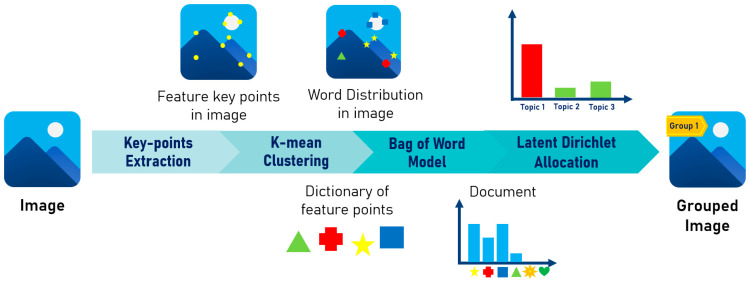
The workflow of topic-modeling-based clustering.

This study proposes a topic-modeling-based approach for clustering and analyzing the thematic content of images using LDA. Since LDA requires textual data as input, the proposed method transforms each image into a document representation composed of visual words before grouping images. The overall workflow of the framework is illustrated in Figure 7, comprising four main stages described below. The training set was used to train the K-means and LDA models to establish the topic-modeling-based clustering pipeline. Subsequently, the test set was processed by fitting it through the K-means and LDA models learned from the training data.

#### 3.4.1. Key-Point Extraction

In the first stage, key points were extracted from all images to capture distinctive local features that represent the underlying structure and content. Two experimental setups were conducted using different feature extraction techniques: (1) Sobel Edge Detection and SIFT, and (2) BGF.

**Figure 8:**
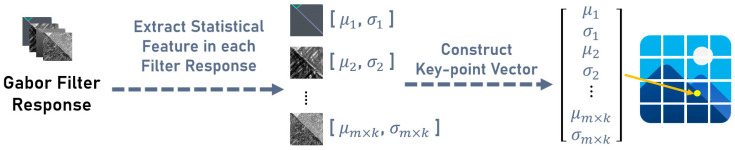
Process of constructing the Gabor filter descriptor.

SIFT is a key-point detection algorithm that locates salient points by computing Difference-of-Gaussians and describes them using gradient orientation histograms over local image regions, providing robustness to changes in scale, rotation, and illumination [18]. In contrast, Block Gabor Filtering (BGF) extracts features by convolving non-overlapping image blocks with Gabor filters at multiple frequencies and orientations, and forms descriptor vectors by concatenating the mean and standard deviation of the resulting filter responses, as illustrated in Figure 8.

In this study, the BGF was configured with 4 scales and 8 orientations, following the experimental settings reported by Yibing Ma et al. [7]. The remaining parameters were assigned as shown in Table 2. For the SIFT-based pipeline, all input images were first processed using Sobel edge detection, after which SIFT was applied to detect key points from the resulting edge-enhanced images. These configurations ensured that both local structural edges (via Sobel and SIFT) and frequency–orientation features (via BGF) were captured effectively.

#### 3.4.2. Feature Clustering and Dictionary Construction

Following key-point detection, each feature descriptor was encoded into a numerical vector. To identify representative visual patterns, the K-means clustering algorithm was applied to these feature descriptors. To determine the optimal number of clusters, the elbow method was applied by analyzing the relationship between the number of clusters (ranging from 2 to 400) and the corresponding inertia values. Based on this analysis, the optimal number of clusters was found to be 59 clusters for SIFT, and 37 clusters for BGF.

Each cluster number was then treated as a visual word. These cluster indices were used to map the key points into representative words, forming the basis of the visual vocabulary or dictionary used in next steps.

#### 3.4.3. Document Representation

After constructing the visual dictionary, each image was represented as a document composed of the visual words derived from the clustering process. The Bag of Words (BoW) model was employed to compute the frequency of each visual word within an image, thus generating a document representation that captures the distribution of visual features across the dataset. This approach adequately converts image features into represented words and aggregates them as new features for inputting into an LDA model. This transformation enabled the use of NLP techniques—specifically, topic model—to uncover the latent semantic structure within the visual data.

#### 3.4.4. Topic Model Using LDA

The resulting document representations were processed using LDA to identify latent themes or topics across the images. The LDA model was trained using the following hyperparameters: α = 0.001, β = 0.001, and maximum iterations = 25.

**Figure 9:**
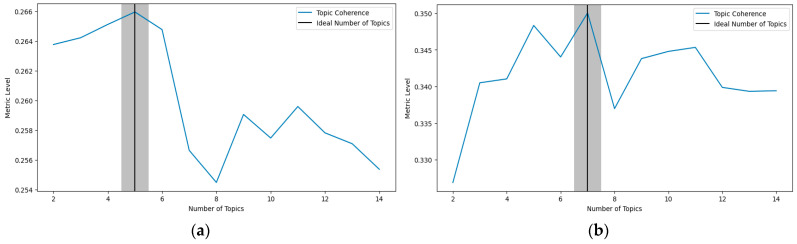
The relationship between the number of topics and the coherence score for SIFT-based (**a**) and BGF-based (**b**) approaches.

The optimal number of topics was determined using the coherence score, which evaluates the semantic consistency of the generated topics. The number of topics corresponding to the highest coherence score was selected as optimal, as illustrated in Figure 9a,b. Based on coherence analysis, the optimal numbers of topics were 5 topics for the SIFT-based experiment, and 7 topics for the BGF-based experiment. Finally, each image was assigned to a topic according to the highest topic proportion in its document-topic distribution, effectively grouping the images to their most representative themes.

### 3.5. Model Training and Selection

Following the image clustering process for both the baseline and proposed methods, the training images within each cluster were used to train several convolutional neural network (CNN) architectures, including AlexNet, GoogLeNet (Inception v1), and the ResNet variants (ResNet18, ResNet34, ResNet50, ResNet101, and ResNet152). To ensure a fair comparison, all CNN architectures were trained under identical experimental settings with a learning rate of 0.001, a batch size of 10, and 15 training epochs. The test images in each cluster were then used to evaluate model performance.

#### 3.5.1. Best-Performing Model Selection

After model evaluation, the architecture achieving the highest test accuracy within each cluster was selected as the best-performing model. In cases where two or more models achieved same test accuracy, the model with the lowest computational complexity was chosen as the preferred model. This strategy ensures an optimal balance between accuracy and efficiency, which is critical for practical deployment in real-world applications.

#### 3.5.2. Model Complexity Considerations

Among the CNN architectures evaluated, model complexity was primarily determined by two factors, (1) the depth of the network and (2) the number of parameters, which directly influence computational cost and memory requirements. A comparative summary of architectural characteristics and complexity levels is presented in Table 3 [19,20,21]. The differences among these models can be outlined as follows:

- AlexNet possesses relatively few layers but a large number of parameters due to its fully connected layers. Despite its simplicity, it requires substantial memory and computation during training;
- GoogLeNet (Inception v1) introduces inception modules and 1 × 1 convolutions, enabling a deeper architecture with significantly fewer parameters compared to AlexNet [20];
- ResNet models progressively increase in depth and parameters from ResNet18 to ResNet152. Their skip connections facilitate effective gradient propagation, allowing successful training of deep networks without performance degradation [21].

Based on network depth and parameter count, ResNet152 is identified as the most complex model, followed sequentially by ResNet101, ResNet50, ResNet34, ResNet18, GoogLeNet, and AlexNet, which is the least complex among the architectures considered. This ranking reflects the trade-off between model capacity and computational demand, guiding model selection for efficiency-aware applications.

### 3.6. Integrating All Best Models

Since multiple models were obtained by selecting the best-performing model within each image cluster, it was necessary to integrate these models into a single unified framework. This integration enabled a fair comparison of performance with models trained on the same test dataset without prior image clustering. Similar to the baseline approach, model integration was required; however, the integration procedure for the proposed method differed slightly while preserving the same underlying concept. The following subsections describe in detail the model integration strategies adopted for both the topic-modeling-based and baseline approaches.

#### 3.6.1. Baseline Integrating Models

As described in Section 3.1, a total of 19 best-performing models were obtained from the individual clusters. To combine these models, two ensemble strategies were employed for the baseline method:

1. Same-cluster model prediction: Each test image was evaluated only by the model corresponding to its respective image cluster;
2. Ensemble voting across all models: Given the large number of models and the lack of weighting factors to indicate the relative importance of each cluster, a simple ensemble voting mechanism was applied across all models to combine their predictions.

Finally, the accuracies obtained from both ensemble strategies were computed and compared with those achieved by the topic-modeling-based method to evaluate relative performance.

#### 3.6.2. Topic-Modeling-Based Model Integration

**Figure 10:**
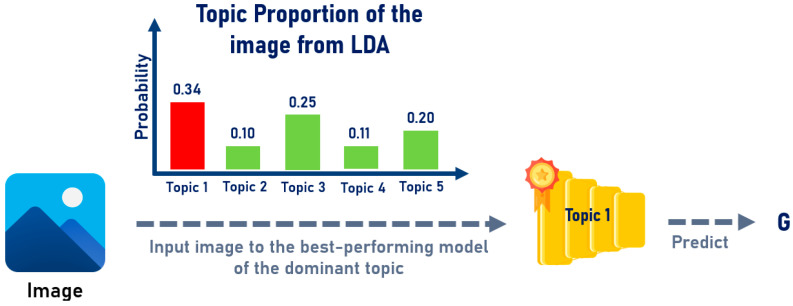
Illustration of the Maximum Proportion Topic Model Integration Method.

**Figure 11:**
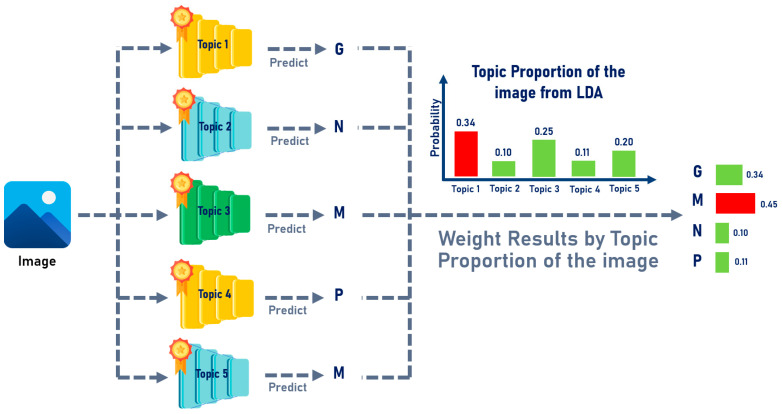
Illustration of the Weight Proportion Topic Model Integration Method.

For the topic-modeling-based method, both the SIFT-based and BGF-based approaches employed the same integration strategy, which was guided by the document–topic distribution of each image. Two model integration techniques were applied, as follows:

1. Maximum Proportion Topic: In this approach, the document–topic distribution of each image was analyzed to identify the topic with the highest proportion. The model corresponding to this dominant topic was then selected to perform the prediction for that image, as illustrated in Figure 10:
2. Weight Proportion Topic: In this method, the best-performing models from all clusters were considered jointly. The topic proportions derived from each image’s document–topic distribution were used as weighting factors to determine the relative contribution of each cluster’s model in the final prediction, as illustrated in Figure 11.

## 4. Results

### 4.1. SIFT-Based

For the proposed method using SIFT combined with the edge detection technique, the number of training and test images in each cluster is summarized in Table 4. It is evident that Cluster 3 has a markedly larger number of training and testing images compared to the other clusters, with 1114 training images and 474 testing images. When calculating the train–test ratio for each cluster, the proportions remain close to the original dataset split of 80:20, indicating balanced distribution across clusters.

The performance of all CNN architectures trained on each cluster is reported in Table 5. The best-performing models for Clusters 1 to 5 are ResNet101 (94.05%), GoogLeNet (89.47%), ResNet101 (97.54%), ResNet152 (98.04%), and ResNet18 (92.48%), respectively. These best models from all clusters were subsequently integrated using the two strategies described in Section 3.6.2.

**Figure 12:**
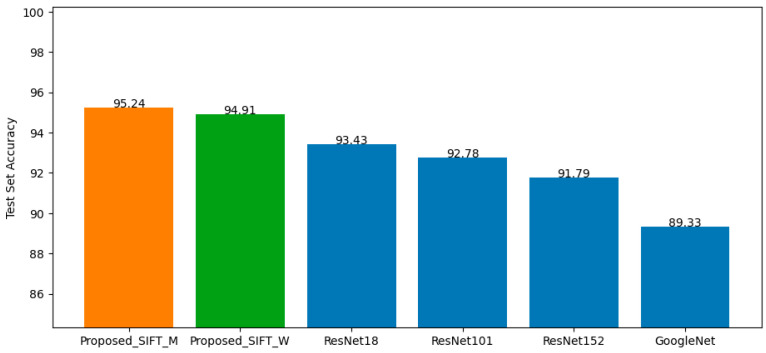
Test accuracy of SIFT-based models using two integration strategies and non-clustering models.

After integrating the best-performing models from all five clusters, the top-performing architectures—GoogLeNet, ResNet18, ResNet101, and ResNet152—were additionally trained on the non-clustered training dataset to compare test accuracy between the proposed clustering-based approach (with both integration strategies) and non-clustered training. The resulting test accuracies were summarized in the bar graph in Figure 12. The results indicate that the proposed method, under both integration strategies, outperforms the non-clustered models. The Maximum Proportion Topic strategy achieves the highest accuracy at 95.24%, demonstrating the effectiveness of topic-guided integration under the SIFT-based setting.

### 4.2. BGF-Based

For the proposed method employing BGF for key-point extraction, the numbers of training and testing images in each cluster are presented in Table 6. Similar to the SIFT-based experiment, Cluster 3 contains the largest number of images, with 617 training images and 174 test images. However, unlike the SIFT-based configuration, the difference between the largest and second-largest clusters is relatively small. The train–test ratios of most clusters remain close to the original 80:20 split, ranging from 78:22 to 85:15.

The test accuracies of all CNN models trained on each cluster are presented in Table 7. The highest-performing models for each cluster are as follows: ResNet152 (95.59%), GoogLeNet (90.91%), ResNet101(94.83%), ResNet101 (95.16%), GoogLeNet (92.59%), GoogLeNet (88.46%), and GoogLeNet (90.38%), respectively. Notably, Clusters 4 and 6 yielded multiple models with identical accuracy; thus, the final selections were made based on model complexity, following the criteria described in Section 3.5.2.

The best models from all clusters were then integrated using the same two strategies applied in the SIFT-based experiment. The models selected for additional training on the non-clustered dataset were GoogLeNet, ResNet101, and ResNet152, as these corresponded to the best models across clusters.

**Figure 13:**
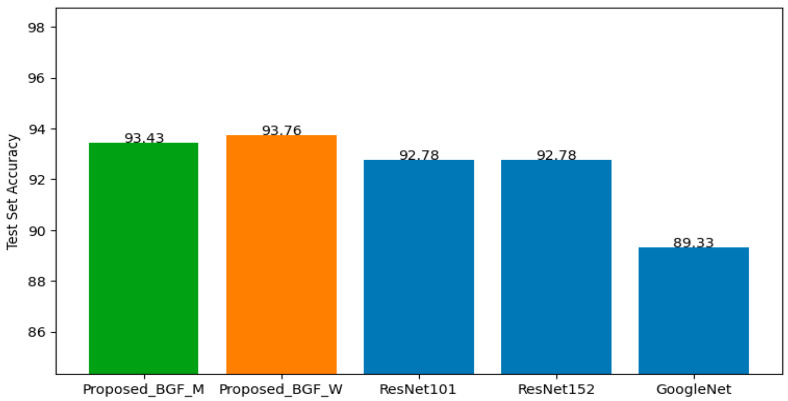
Test accuracy of BGF-based models using two integration strategies and non-clustering models.

The results for both the non-clustered baseline models and the proposed integrated models are illustrated in Figure 13. Consistent with the SIFT-based findings, the proposed method—under both integration strategies—outperforms the non-clustered training approach. The Weight Proportion Topic strategy achieves the highest accuracy at 93.76%, indicating its suitability for the BGF-based experiment.

### 4.3. Baseline

**Figure 14:**
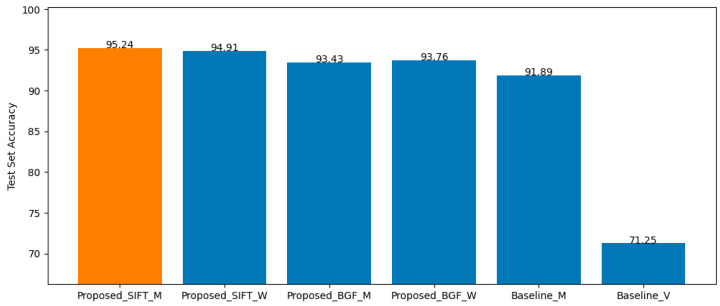
Test accuracy of SIFT-based, BGF-based, and baseline clustering models.

For the baseline method, all 19 best-performing models from their respective clusters were integrated using two strategies: Same-Cluster Prediction (Baseline_M) and Ensemble Voting (Baseline_V). The resulting test accuracies are 91.89% and 71.25%, respectively. These results, together with the SIFT-based and BGF-based findings presented in Section 4.1 and Section 4.2, are compared in the bar graph shown in Figure 14.

The comparison clearly demonstrates that the proposed method—both SIFT-based and BGF-based—outperforms the baseline under both Same-Cluster Prediction and Ensemble Voting strategies. Among all evaluated models, the SIFT-based approach using the Maximum Proportion Topic integration achieves the highest overall performance.

## 5. Discussion

Overall, the results demonstrate that topic modeling serves as an effective preprocessing framework for image classification, enhancing both clustering quality and downstream predictive performance. Based on the findings presented in Section 4.1, Section 4.2 and Section 4.3, integrating topic modeling with cluster-specific CNN training significantly improves classification performance compared with traditional training on non-clustered datasets. Moreover, both the SIFT-based and BGF-based variants of the proposed clustering method achieved higher test accuracies than the non-clustered approaches, indicating that the latent semantic structure extracted through LDA contributes meaningfully to organizing the image space into more homogeneous and discriminative subsets.

The SIFT-based approach yielded the highest overall performance, achieving a peak accuracy of 95.24% under the Maximum Proportion Topic strategy. This superiority may be attributed to the effectiveness of SIFT and gradient-based key points in capturing salient local structures that align well with the semantic topics generated by LDA, thereby supporting more coherent cluster formation. Although the BGF-based approach achieved a slightly lower maximum accuracy of 93.76%, it still outperformed all non-clustered training models. While BGF effectively encodes frequency–orientation patterns, its descriptors may be less discriminative than those derived from SIFT for the dataset used in this study.

The comparison with baseline methods further illustrates the advantages of topic-guided clustering. One limitation of baseline clustering approaches that do not incorporate topic modeling methods is that they may produce an excessive number of clusters. This can result in some clusters containing too few images for effective training, or even lacking samples from certain classes altogether, which inevitably hinders the training and reliability of cluster-specific models. The Same-Cluster Prediction baseline achieved an accuracy of 91.89%, whereas Ensemble Voting performed substantially worse at 71.25%, likely due to the absence of weighting mechanisms and the heterogeneity of image content across clusters. These findings underscore the importance of semantic relevance when aggregating model predictions. The proposed integration strategies—particularly the Maximum Proportion Topic and Weight Proportion Topic methods—provide more principled mechanisms for model integration, resulting in consistently higher accuracies.

An additional practical consideration is the computational cost of the preprocessing stage. In this study, all preprocessing was performed on a computer equipped with an Intel Core i7-13620H CPU and 32 GB of RAM. The preprocessing time was 81 min 44.7 s for the SIFT-based approach and 80 min 4.8 s for the BGF-based approach, largely due to training multiple models (5 × 7 CNN models for SIFT-based clustering and 7 × 7 models for BGF-based clustering). By comparison, non-clustered CNN models required approximately 5 min 59.1 s to 25 min 14.1 s for training. Although this increases overall computational time, the preprocessing stage substantially improves clustering quality and classification accuracy, thereby enhancing downstream performance. This trade-off underscores the benefit of investing computational resources at the preprocessing stage.

An interesting observation is the variation in cluster sizes across the experiments, especially in the SIFT-based scenario, where one cluster was substantially larger than the others. Despite this imbalance, the train–test proportion of each cluster remained close to the original 80:20 split, and classification performance did not degrade. This suggests that the topic modeling process produced stable and meaningful partitions. The success of the integration strategies further indicates that topic distributions not only guide clustering but also serve as informative indicators for model selection during inference.

Regarding generalizability, the proposed framework is not specific to brain tumor imaging but is applicable to image classification tasks characterized by substantial intra-class visual variability. By leveraging topic modeling to partition images into semantically coherent subsets, the approach enables CNNs to specialize in more homogeneous data distributions. This mechanism is expected to be beneficial for other image domains, such as natural scenes or medical imaging tasks with diverse visual patterns. However, the effectiveness of the framework may depend on the chosen feature representation and dataset characteristics, and further validation on additional image classes is required to confirm its generalizability.

Beyond performance considerations, the deployment of AI-based methods in medical imaging also raises important concerns regarding reliability, interpretability, and clinical responsibility. Recent studies have highlighted risks associated with AI-driven diagnostic systems, including limited generalizability across datasets, sensitivity to data imbalance, and the potential for over-reliance in clinical decision-making [22,23,24]. In this context, the proposed framework is not intended to function as an autonomous diagnostic system, but rather as a supportive or assistive tool that assists clinicians by organizing heterogeneous image data into semantically meaningful clusters and improving downstream model performance. Final diagnostic decisions remain the responsibility of medical professionals, with the proposed method serving to enhance interpretability and efficiency during image analysis. This positioning aligns with current recommendations that emphasize human–AI collaboration to ensure safe and responsible deployment of AI in clinical practice.

## 6. Conclusions

This study introduced a topic-modeling-based framework for image classification in which images are transformed into visual documents using SIFT or BGF descriptors and subsequently grouped into latent topics via LDA. Cluster-specific CNN models were trained and integrated using two topic-guided strategies. Experimental results show that both variants of the proposed method significantly outperform baseline approaches that rely solely on cluster labels or unweighted ensemble voting. The highest accuracy of 95.24% was achieved by the SIFT-based method with the Maximum Proportion Topic strategy, while the BGF-based method reached 93.76% using the Weight Proportion Topic strategy.

These findings confirm that incorporating semantic structure through the topic model enables more effective learning within the image classification pipeline. By training each CNN on a semantically coherent subset of images and informing predictions through topic distributions, the proposed method achieves superior predictive performance compared with traditional non-clustered CNN training.

Nevertheless, some limitations remain. The approach is computationally expensive due to the need to train multiple models, and cluster quality is sensitive to the choice of key-point extraction methods. In addition, model selection was based solely on the highest test accuracy obtained from a single training run; therefore, statistical significance testing (e.g., *p*-values) was not conducted. Future work may explore alternative feature representations, such as deep visual embeddings, as well as transformer-based architectures for both clustering and classification. In addition, further research is needed to improve LDA-based image clustering by addressing the issue of data imbalance among topic clusters, where unequal numbers of images may affect cluster stability and downstream model performance. Another important direction is to overcome the limitation of standard bag of visual-word representations, which ignore spatial layout information. The incorporation of spatially aware representations, such as Spatial Pyramid Matching or Vector of Locally Aggregated Descriptors (VLAD), could preserve spatial structure and provide more informative feature representations. Furthermore, integrating automated model selection and extending the evaluation to larger-scale datasets may further enhance the generality and robustness of the proposed framework.

Overall, this research enhances medical image classification performance by integrating topic modeling with deep learning. Topic modeling provides more informative and semantically coherent clustering than classical clustering methods, leading to improved classification accuracy. Although the framework requires additional computational effort during preprocessing, the resulting performance gains justify this overhead.

## Figures and Tables

**Table 1:** Details of the training and testing images in each cluster from the baseline clustering.

Cluster Number	The Number of Training Images	Non-Available Class in Train Set	The Number of Testing Images	Non-Available Class in Test Set
37	150	-	30	-
4	116	-	33	-
8	114	-	26	-
19	103	-	21	N
44	96	-	26	N
47	76	-	32	M
3	75	N	16	N
26	73	-	15	N
27	71	N	27	-
36	69	-	15	P
17	68	-	19	N
11	67	-	19	G
29	67	-	21	-
38	63	N	20	N
0	60	N	16	N, P
13	59	-	23	-
9	58	-	15	-
7	53	-	18	-
21	52	-	15	P

**Table 2:** Parameter settings for the BGF algorithm.

Parameters	Value Setting
σ or scale Interval	[2, 6]
θ or orientation Interval	[0,π]
Phase Shift φ	0
Wavelength λ	π/4
Spatial Aspect Ratio γ	1

**Table 3:** The number of layers and parameters in each CNN model.

Model	Number of Layers	Number of Parameters
AlexNet	8	62 million
GoogLeNet	22	6.8 million
ResNet18	18	11.69 million
ResNet34	34	21.80 million
ResNet50	50	25.58 million
ResNet101	101	44.55 million
ResNet152	152	60.19 million

**Table 4:** Details of training and testing images in each cluster from SIFT-based clustering.

Experimental Clusters	Number of Training Images	Number of Testing Images	Train–Test Ratio
Topic 1	399	84	83:17
Topic 2	211	57	79:21
Topic 3	1114	284	80:20
Topic 4	246	51	83:17
Topic 5	462	133	78:22

**Table 5:** Accuracies of CNN models in each cluster from SIFT-based clustering.

Experimental Clusters	AlexNet	GoogleNet	ResNet18	ResNet34	ResNet50	ResNet101	ResNet152
Topic 1	60.71	88.10	72.38	85.71	80.95	94.05	83.33
Topic 2	57.89	89.47	66.67	87.72	87.72	82.46	80.70
Topic 3	64.08	92.96	86.62	90.14	90.85	97.54	90.49
Topic 4	74.51	96.08	92.16	94.12	92.16	92.16	98.04
Topic 5	54.89	86.47	92.48	91.73	84.96	90.98	66.17

**Table 6:** Details of training and testing images in each cluster from BGF-based clustering.

Experimental Clusters	Number of Training Images	Number of Testing Images	Train–Test Ratio
Topic 1	535	136	80:20
Topic 2	553	132	81:19
Topic 3	617	174	78:22
Topic 4	238	62	79:21
Topic 5	155	27	85:15
Topic 6	116	26	82:18
Topic 7	218	52	81:19

**Table 7:** Accuracies of CNN models in each cluster from BGF-based clustering.

Experimental Clusters	AlexNet	GoogleNet	ResNet18	ResNet34	ResNet50	ResNet101	ResNet152
Topic 1	33.82	91.18	86.76	85.29	87.50	91.91	95.59
Topic 2	73.48	90.91	87.88	87.12	84.09	81.06	69.70
Topic 3	64.94	70.11	93.1	89.08	93.10	94.83	87.36
Topic 4	56.45	91.94	90.32	70.97	91.94	95.16	95.16
Topic 5	55.56	92.59	29.63	85.19	77.78	81.48	77.78
Topic 6	42.31	88.46	76.92	73.08	88.46	88.46	46.15
Topic 7	61.54	90.38	86.54	69.23	80.77	78.85	84.62

## Data Availability

The original data presented in the study are openly available in Kaggle at https://www.kaggle.com/datasets/thomasdubail/brain-tumors-256x256 (accessed on 20 April 2025).
